# Automated acute ischemic stroke lesion delineation based on apparent diffusion coefficient thresholds

**DOI:** 10.3389/fneur.2023.1203241

**Published:** 2023-07-27

**Authors:** Vitus Gosch, Kersten Villringer, Ivana Galinovic, Ramanan Ganeshan, Sophie K. Piper, Jochen B. Fiebach, Ahmed Khalil

**Affiliations:** ^1^Center for Stroke Research Berlin, Charité – Universitätsmedizin Berlin, Corporate Member of Freie Universität Berlin and Humboldt-Universität zu Berlin, Berlin, Germany; ^2^Institute of Medical Informatics, Charité – Universitätsmedizin Berlin, Corporate Member of Freie Universität Berlin and Humboldt-Universität zu Berlin, Berlin, Germany; ^3^Institute of Biometry and Clinical Epidemiology, Charité – Universitätsmedizin Berlin, Corporate Member of Freie Universität Berlin and Humboldt-Universität zu Berlin, Berlin, Germany

**Keywords:** acute ischemic stroke, diffusion-weighted imaging, automated lesion delineation, apparent diffusion coefficient, MRI analysis software

## Abstract

**Purpose:**

Automated lesion segmentation is increasingly used in acute ischemic stroke magnetic resonance imaging (MRI). We explored in detail the performance of apparent diffusion coefficient (ADC) thresholding for delineating baseline diffusion-weighted imaging (DWI) lesions.

**Methods:**

Retrospective, exploratory analysis of the prospective observational single-center 1000Plus study from September 2008 to June 2013 (clinicaltrials.org; NCT00715533). We built a fully automated lesion segmentation algorithm using a fixed ADC threshold (≤620 × 10–6 mm^2^/s) to delineate the baseline DWI lesion and analyzed its performance compared to manual assessments. Diagnostic capabilities of best possible ADC thresholds were investigated using receiver operating characteristic curves. Influential patient factors on ADC thresholding techniques’ performance were studied by conducting multiple linear regression.

**Results:**

108 acute ischemic stroke patients were selected for analysis. The median Dice coefficient for the algorithm was 0.43 (IQR 0.20–0.64). Mean ADC values in the DWI lesion (*β* = −0.68, *p* < 0.001) and DWI lesion volumes (*β* = 0.29, *p* < 0.001) predicted performance. Optimal individual ADC thresholds differed between subjects with a median of ≤691 × 10^−6^ mm^2^/s (IQR ≤660–750 × 10^−6^ mm^2^/s). Mean ADC values in the DWI lesion (*β* = −0.96, *p* < 0.001) and mean ADC values in the brain parenchyma (*β* = 0.24, *p* < 0.001) were associated with the performance of individual thresholds.

**Conclusion:**

The performance of ADC thresholds for delineating acute stroke lesions varies substantially between patients. It is influenced by factors such as lesion size as well as lesion and parenchymal ADC values. Considering the inherent noisiness of ADC maps, ADC threshold-based automated delineation of very small lesions is not reliable.

## 1. Introduction

Recent research further advancing the time window for mechanical thrombectomy and intravenous thrombolysis highlights the importance of tissue-based criteria in acute ischemic stroke (AIS) therapy (1, 2). Quantifying the ischemic core has been of central importance in trials using advanced imaging to guide stroke treatment (3, 4).

In clinical practice, visual assessment of diffusion-weighted imaging (DWI) lesions and computed tomography perfusion (CTP) thresholding are used for the estimation of the ischemic core, with DWI being more sensitive (5, 6). The visual assessment of DWI lesions as an estimation for unsalvageable tissue is subjective and lacks a clinically available gold standard (5). Efforts have thus been made to make the identification of the ischemic core using the apparent diffusion coefficient (ADC) more objective. As an absolute diffusivity measurement, the ADC appears well-suited for thresholding techniques across locations and vendors (7). Furthermore, the ADC can be used to further stratify the ischemic core into reversible and irreversible components (8, 9).

With rising demand for infarct quantification, automated lesion segmentation software is increasingly used in trials and clinical practice (10). For algorithms based on MRI, most commercially available lesion segmentation algorithms use absolute ADC thresholds for the determination of the ischemic lesion (10). The two most widely used algorithms RAPID^®^ (iSchemaView) and Olea Sphere^®^ (Olea Medical), use fixed or user-adjustable generalized absolute ADC thresholds (11–13). The threshold of ≤620 × 10–6 mm^2^/s currently used in RAPID^®^ and also frequently applied using Olea Sphere^®^ was initially proposed in 2015 by Purushotham et al. (8, 14). Their study determined an ADC threshold for distinguishing between tissue inside the DWI lesion destined to infarct and reversible regions. The threshold was volumetrically verified on 14 patients using the manually delineated DWI lesion as ground truth (8).

While these algorithms are already used in clinical practice and were implemented in many multicenter studies, the underlying assumptions behind this ADC threshold have not yet been independently replicated (3, 4, 10). This ADC threshold was determined on a small sample size and only volumetrically verified, lacking detailed spatial analysis (8). Differences in automated and manual lesion delineations and factors affecting the performance of ADC-based lesion segmentation algorithms remain scarcely investigated.

In this study, we explored in detail the performance of the widely used ADC threshold (≤620 × 10–6 mm^2^/s) for delineating baseline DWI lesions and the performance of ADC thresholds in general. To that end, we built a lesion segmentation algorithm that used ADC thresholding and compared its performance to manual DWI lesion delineation in a large cohort of acute ischemic stroke patients. The algorithm was intended to replicate commercially available DWI delineation software, supplemented with artifact reduction techniques adopted from previous research (8, 15) in order to gain insights into the performance of ADC thresholds for automated DWI lesion delineation.

## 2. Materials and methods

### 2.1. Participants

All patients participated in the prospective observational 1000Plus study from September 2008 to June 2013 (clinicaltrials.org NCT00715533). The single-center study performed by the Center for Stroke Research Berlin acquired MRI data from 1472 patients presenting to the emergency room with a clinical diagnosis of an acute cerebrovascular event (16). The study design was approved by the institutional review board of the Charité Universitätsmedizin, Berlin (EA4/026/08). The study was carried out in accordance with The Code of Ethics of the World Medical Association (Declaration of Helsinki). Written informed consent was obtained from all patients.

**Figure 1:**
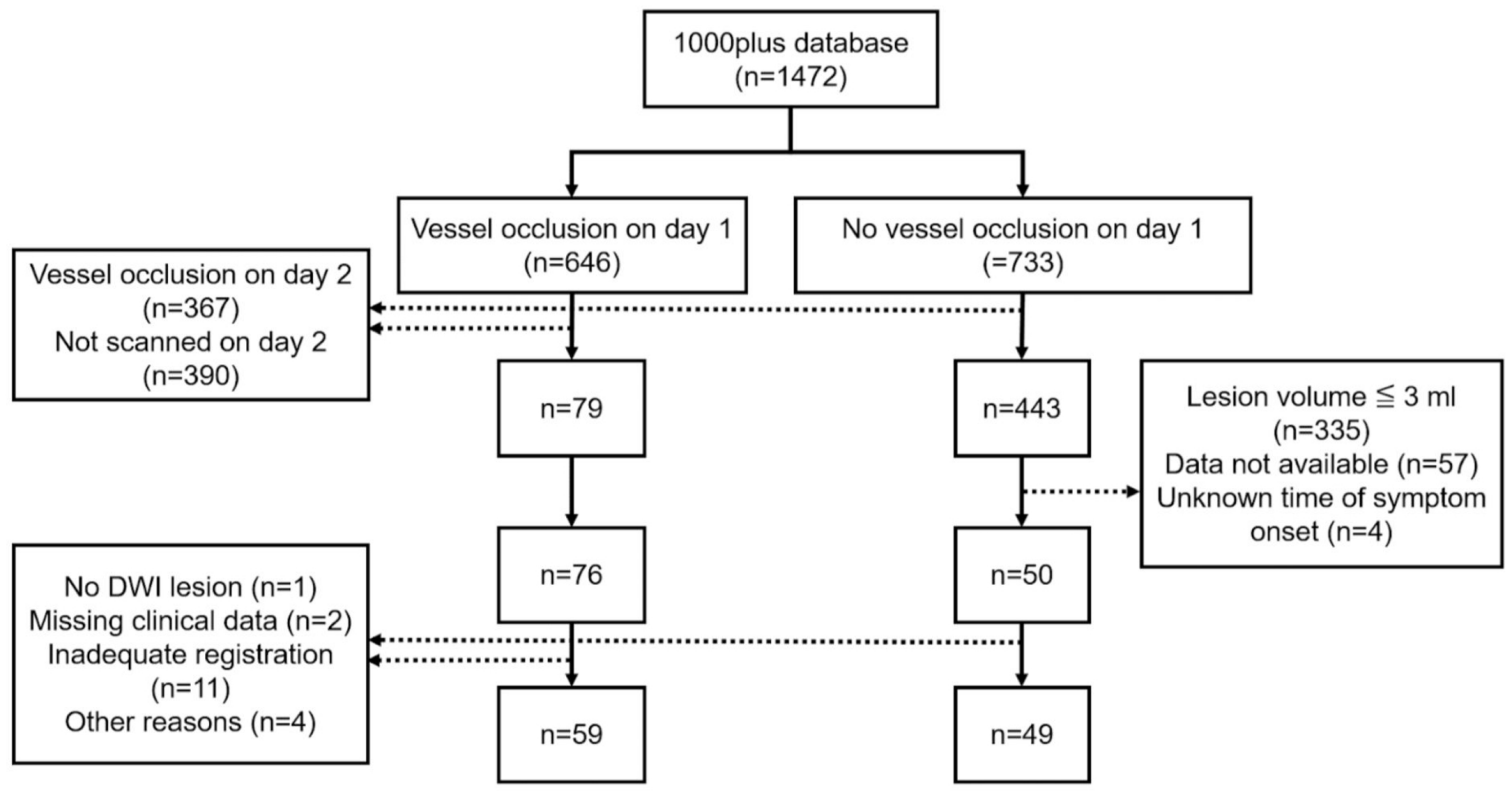
Inclusion flowchart.

A subset of 126 patients was selected for retrospective analysis. Inclusion criteria included available DWI data, either a vessel occlusion on day 1 with vessel recanalization on day 2 or no vessel occlusion on day 1, as well as the known time of symptom onset. Vessel recanalization on day 2 was required to investigate the degree to which automatically delineated DWI lesions on day 1 reflect final infarct volume (assessed between days 3 and 5 using FLAIR) (17). In the group without vessel occlusion on day 1, we excluded very small strokes (≦3 mL in volume) because we considered that these would not be appropriate for automated threshold-based delineation. Following the exclusion of 18 patients after data processing 108 patients were selected for analysis (Figure 1).

### 2.2. Imaging protocol

Imaging was performed on a 3T MRI scanner (Tim Trio; Siemens AG, Erlangen, Germany). DWI was performed with a spin-echo echo-planar imaging sequence with a ‘b’ value of 1000 s/mm^2^ (TR/TE = 7600/93 ms, matrix = 192 × 192, field of view = 230 mm, slice thickness = 2.5 mm, slice gap = 0 mm, number of slices = 50, acquisition time = 2 min 11 s). The study protocol also included T2*-weighted images, time-of-flight magnetic resonance angiography (TOF-MRA), and fluid-attenuated inversion recovery (FLAIR) images (16). All participants were scanned relative to the time of stroke symptom onset (TOO) on day 1 (within 24 h) and day 2 (24–48 h). In 78 patients, follow-up imaging data obtained around the fifth day of admission (range: day 3–7, mean 4.8 days) was available.

### 2.3. Image preprocessing

All images were first converted from DICOM to NIfTI format using dcm2nii software (18). Brain extraction was performed on b0, trace (*b* = 1000), and ADC map images using BET (Brain Extraction Tool) (19). The brain-extracted b0 images were registered to MNI152 T1-weighted 2 mm isovoxel standard space using three-dimensional diffeomorphic symmetric normalization (using *antsRegistrationSyNQuick*) (20–24). Registered images were checked visually and unsatisfactory registrations were excluded. Resulting transformation matrices were used for mapping of ADC map and trace images to MNI152 standard space using *antsApplyTransforms* (20–24). Tissue segmentation of cerebrospinal fluid (CSF) and brain parenchyma was conducted on b0 images using FMRIB’s Automated Segmentation Tool (25). Baseline DWI lesions and follow-up FLAIR lesions were delineated by a group of stroke imaging researchers using MRIcro and supervised by either an experienced radiology resident, a board-certified neuroradiologist, or a radiologist (26). DWI ROIs were mapped nonlinearly to MNI152 standard space using *antsApplyTransforms* (20–23).

### 2.4. Input to automated lesion delineation algorithm

MR imaging data obtained at admission within 24 h of symptom onset were used. After image preprocessing, the brain extracted and registered b0, trace, and ADC map images were read by the algorithm.

### 2.5. Automated lesion delineation

**Figure 2:**
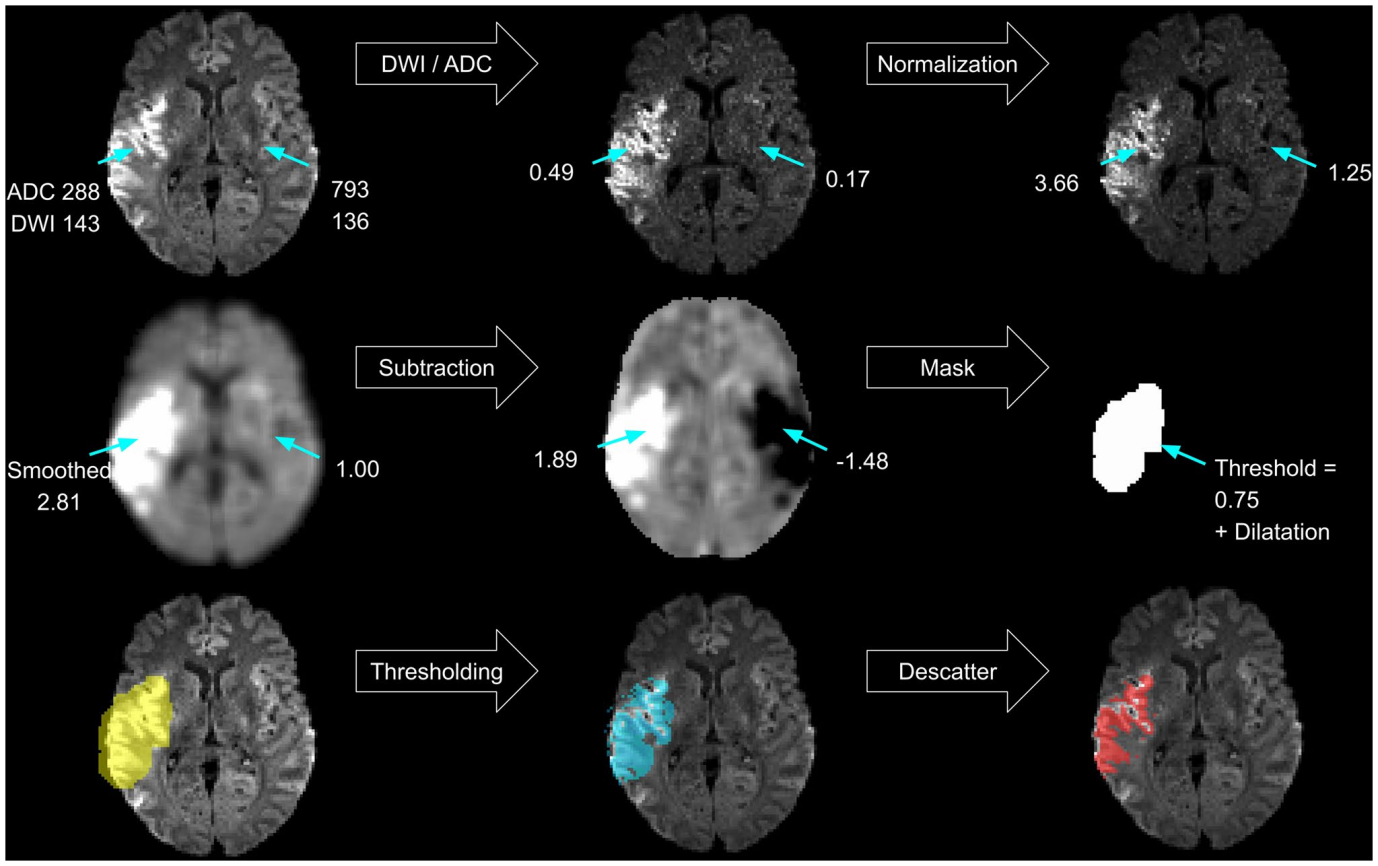
Algorithm. Schematic steps performed within the thresholding algorithm.

A threshold-based lesion segmentation algorithm was developed using the FSL software package (27–29). The approach solely relies on the acquired DWI sequence (the trace and ADC) and outputs a lesion mask that aims to match the expert lesion. It uses artifact reduction techniques known from previous research and uses ADC thresholds already implemented in commercially available software (8, 15). First, the algorithm localizes the infarct and creates a primary lesion mask (see Figure 2). Artifact reduction and lesion contrast enhancement are achieved by dividing the trace image by the ADC map. Resulting relative voxel values inherit greater contrast between healthy and ischemic tissue. To assure comparability between subjects and brain regions, normalization of relative values is performed in every slice separately by dividing the voxel’s signal intensity by the average voxel value of the whole slice. Consecutively, smoothing using an isotropic 5 voxel Gaussian kernel is performed. Mirroring along the *x*-axis and subtraction of the contralateral hemisphere further reduces artifacts. A lower threshold of 0.75 image intensity is applied to the resulting processed image based on testing lesion coverage on a subset of 5 randomly chosen subjects. Partial dilatation completes the mask creation. Finally, the delineated infarct region includes areas within the mask with ADC values between 200 and 620×10^−6^ mm^2^/s (8). Removal of scattered trace-hypointense voxels is achieved by removing voxels with trace intensities below the 95th percentile (Figure 2).

### 2.6. Statistical analysis

Statistical analysis of this retrospective, exploratory study was performed using R Studio Version 2022.07.1 + 554 (30). The Dice coefficient


$$D=\frac{2TP}{2TP+FP+FN}$$

was used to study spatial overlap between the automated, and the manual delineated ROIs (31). Volumetric agreement between manual and automated DWI lesion volumes as well as between initial lesion and follow-up FLAIR lesion volumes was investigated using Bland–Altman analysis (32). The correlation of manual and automated delineated lesion volumes was assessed by calculating Pearson’s correlation coefficient (33). A whole-brain ROC analysis was conducted in every subject using the *fslmaths* function of the FSL software package to determine the optimal ADC threshold for distinguishing infarcted from healthy tissue (28, 29). Therefore, voxel-wise analysis was performed between ADC values of manually delineated day one DWI lesions and the whole brain parenchyma ADC map. A second analysis used the same ground truth, but a different ADC map centered on the DWI lesion. This was achieved by dilating the DWI lesion using the *fslroi* function from the FSL toolbox and applying the resulting mask to the ADC map (29). ROC analysis output was pooled across subjects to identify a generalized threshold. The Youden index


J=sensitivity+specificity−1

was used to determine the best possible threshold (34). Multivariate and univariate linear regression was used to investigate whether initial DWI lesion size, DWI lesion ADC value, mean ADC in the brain parenchyma, age or TOO would be associated with the performance of the segmentation algorithm itself and the diagnostic capability of the ADC threshold found in the patient. As the performance of the algorithm was measured in spatial overlap, the Dice coefficient was the response variable in the first model. As diagnostic capability was assessed with the Youden index (YI) it represented the response variable in the second model. Assumptions for using linear models were checked, assessing skewness, kurtosis, link function and heteroscedasticity with the R packages “lmtest,” “gvlma” and “corrplot” (35–37). Visualization was accomplished using the R package “ggplot2” (38).

The image processing scripts, segmentation algorithm, and statistical analysis scripts are openly accessible.

## 3. Results

### 3.1. Baseline characteristics

The baseline characteristics of the 108 patients analyzed are summarized in Table 1.

**Table 1:** Patient characteristics.

Variable	Descriptive statistic
Patients (female)	108 (♀ = 38)
Mean age in years (±SD)	68 (±14)
Hypertension, n (%)	77 (71%)
Diabetes mellitus, n (%)	21 (19%)
Hyperlipidemia, n (%)	53 (49%)
Median time from onset to MRI*	247 min (IQR 108–737 min)
With vessel occlusion, n (%)	76 (79%)
Median Wahlund score	5 (IQR: 4–8)
Median NIHSS† at admission	3 (IQR: 1–6)
Type of infarction, n (%)	
Territorial	99 (91.7%)
Lacunar	5 (4.6%)
Borderzone	4 (3.7%)
Infratentorial infarction, n (%)	16 (15%)

*MRI, magnetic resonance imaging; †NIHSS, National Institutes of Health Stroke Scale.

### 3.2. Infarct characteristics within 24 h of symptom onset

**Figure 3:**
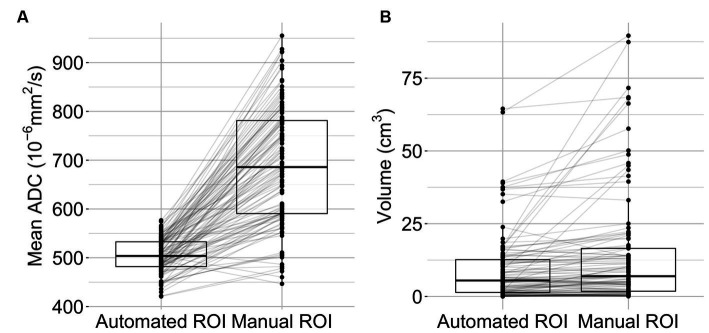
Lesion characteristics. Mean lesion ADC values **(A)** and lesion volumes **(B)** are displayed as boxplots with individual data points (*n* = 108). The middle bars represent the median, the box indicates the lower (Q1) and upper (Q3) quartiles. Lines connect observations within one subject.

After applying the segmentation algorithm to imaging data acquired within 24 h of symptom onset (median 247 min, IQR 108–737 min), the resulting delineations were compared to manually delineated ROIs. The median ADC value in the automated ROIs was 504 × 10^−6^ mm^2^/s (IQR 421–533 × 10^−6^ mm^2^/s) compared to 686 × 10^−6^ mm^2^/s (IQR 591–780 × 10^−6^ mm^2^/s) in the manual ROIs (see Figure 3A). Automated ROIs had a median lesion size of 5.5 mL (IQR 1.4–12.7 mL), and the median volume of manual ROIs was 6.9 mL (IQR 1.8–16.5 mL) (Figure 3B).

### 3.3. Volumetric agreement and spatial overlap

**Figure 4:**
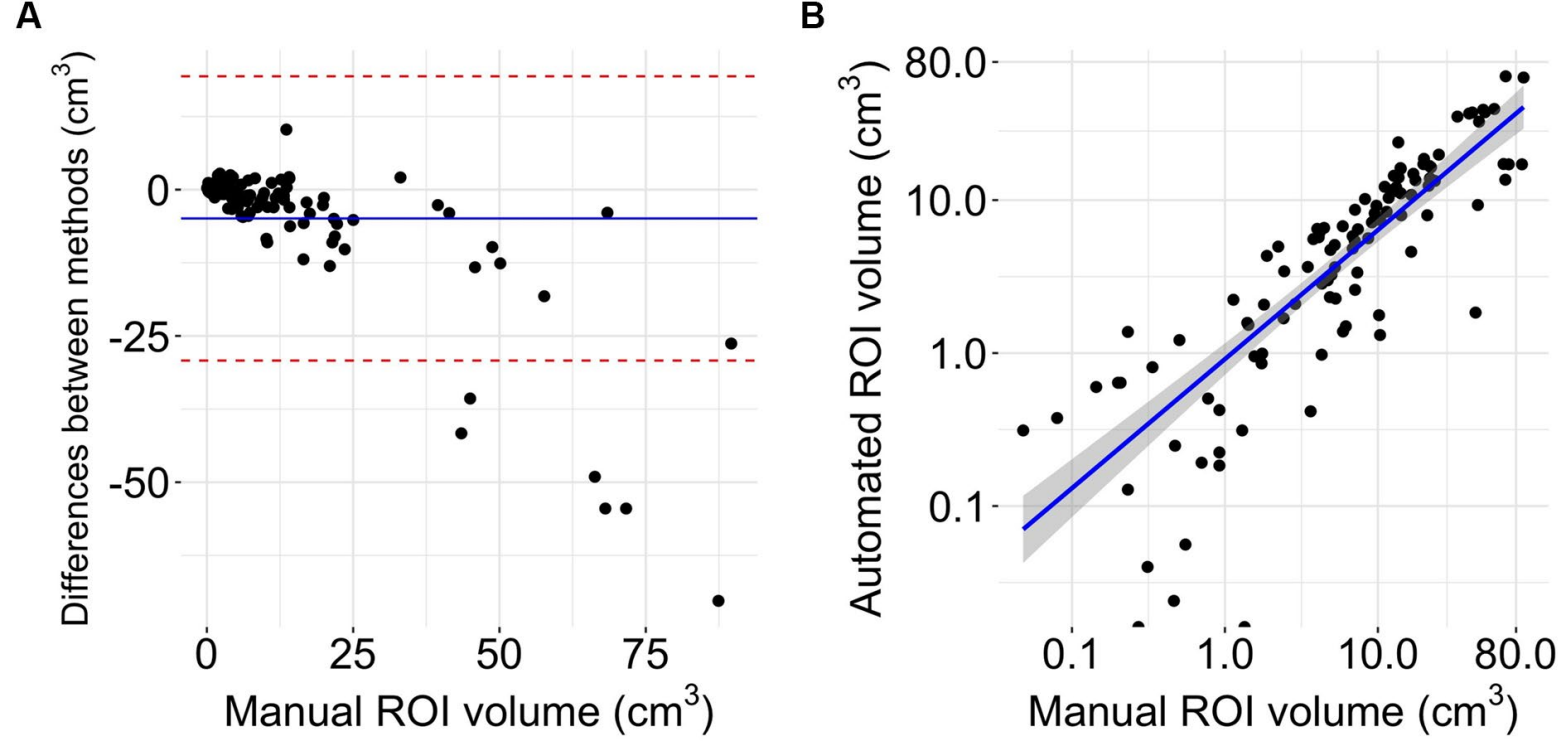
Volumetrics. **(A)** Bland Altman plot of volume differences (*n* = 108). The *Y*-axis shows the difference between automated and manual delineations (manual-automated). The *X*-axis represents the reference method (32). The blue line displays mean difference, the red lines indicate limits of agreement from −1.96 to +1.96 standard deviations of mean difference. **(B)** Logarithmic scaled scatterplot comparing manual and automated ROI sizes with blue line of regression and gray 95% confidence interval (*n* = 108).

**Figure 5:**
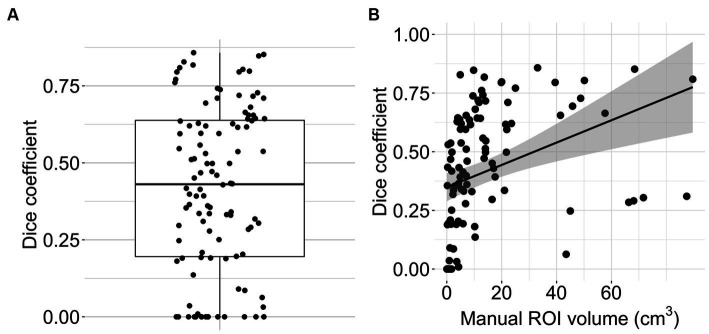
Algorithm performance. **(A)** Boxplot with overlain dot plot displaying Dice coefficients of the automated lesion delineations (*n* = 108). **(B)** Scatter plot showing the relationship between manual ROI volume and Dice coefficient for the automated segmentation (*n* = 108). The line represents smoothed conditional means.

The Bland Altman analysis showed a mean bias of −4.92 mL with an upper limit of agreement of 19.36 mL and a lower limit of agreement of −29.21 mL (Figure 4A). The volumes of automated and manual ROIs correlated strongly, *r*(106) = 0.79 (CI = 0.7–0.85), *p*-value < 0.0001 (Figure 4B). Linear regression had a slope of 0.49 (adjusted *R*^2^ = 0.62, *p*-value < 0.0001). In two outlier subjects, the algorithm did not detect an infarction. The median Dice coefficient for the automated segmentation was 0.43 (IQR 0.20–0.64) (Figure 5A). No spatial overlap was seen in 15 lesions with low volume (manually delineated lesion size IQR: 0.2–0.8 mL, median 0.46 mL) (see Figure 5B).

### 3.4. ROC analysis

**Figure 6:**
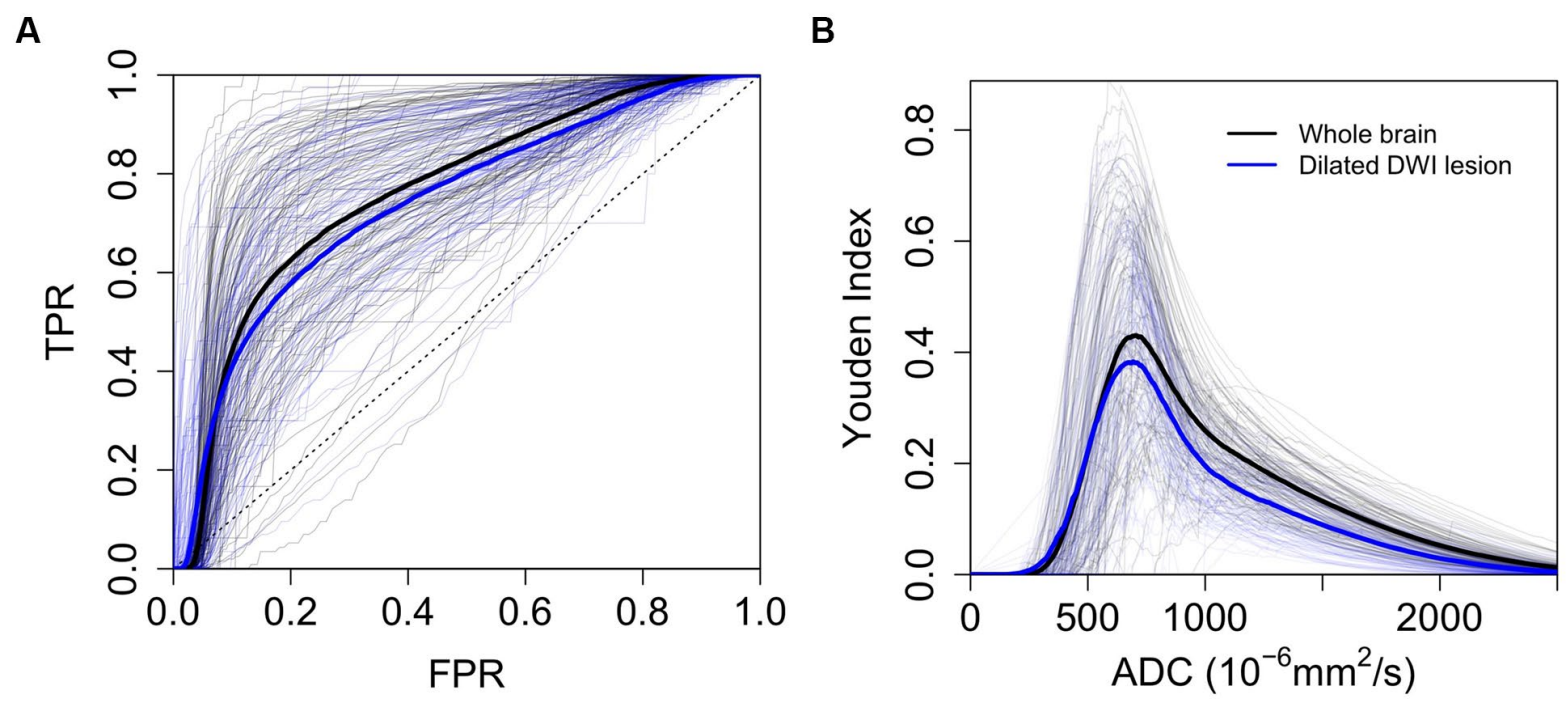
ROC Analysis. **(A)** ROC analysis to determine the optimal threshold for automated delineation of ischemic lesions (*n* = 108). **(B)** Youden index plotted against individual and pooled ADC thresholds (*n* = 108). Thick lines represent mean ROC curves across patients (blue for dilated DWI lesion and black for whole-brain parenchyma). TPR (true positive rate), FPR (false positive rate).

The optimal generalized threshold to match the expert ischemic lesion delineation in the brain parenchyma using pooled ROC analysis was an ADC ≤704 × 10^−6^ mm^2^/s (sensitivity 65% and specificity 77%, AUC 0.76). Narrowing down the search area by a median of 96% (IQR 0.93–0.97) to the vicinity of the infarct resulted in an optimal pooled threshold of ≤693 × 10^−6^ mm^2^/s (sensitivity 63% and specificity 75%, AUC 0.74). In both cases, individual ROC curves differed substantially from those generated using pooled data (see Figure 6). The individual whole-brain ROC analysis determined thresholds with a median of 691 × 10^−6^ mm^2^/s (IQR 660–750 × 10^−6^ mm^2^/s; sensitivity median 75%, IQR 64–86%; specificity median 80%, IQR 73–83%).

### 3.5. Linear model

Sixteen patients with unknown time from symptom onset to imaging were excluded from this analysis. At first, predictors of the performance of the segmentation algorithm were assessed. The overall regression was statistically significant (adjusted R-squared = 0.54, *F*(5, 86) = 22.03, *p* < 0.001). The mean ADC value in the DWI lesion (*β* = −0.65, [−0.80 – −0.50], *p* < 0.001) and the DWI lesion volume (*β* = 0.28, [0.13–0.42], *p* < 0.001) were significantly associated with the Dice coefficient. The mean ADC values in the brain parenchyma (*β* = 0.16, [−0.03–0.35], *p* = 0.09), time from onset to imaging (*β* = 0.01, [−0.13–0.16], *p* = 0.84) and patient age (*β* = −0.17, [−0.36–0.01], *p* = 0.07) were not.

Secondly, variables possibly impacting the identification of individual ADC thresholds as determined by ROC analysis (compare Figure 6) were analyzed. The dependent variable was the Youden index (YI). The overall regression was statistically significant (adjusted R-squared = 0.87, *F*(5, 86) = 118.7, *p* < 0.001). Significant predictors for the Youden index were mean ADC values in the DWI lesion (*β* = −0.96, [−1.04 – −0.87], *p* < 0.001), mean ADC values in the brain parenchyma (*β* = 0.28, [0.18–0.38], *p* < 0.001), DWI lesion volume (*β* = −0.11, [−0.18 – −0.03], *p* = 0.006) and patient age (*β* = −0.14, [−0.25 – −0.04], *p* = 0.006). Time from onset to imaging (*β* = 0.05, [−0.03–0.12], *p* = 0.20) did not show a significant association. Visualization of the results of the regression analysis and additional univariate models can be accessed in the Supplementary Figures S1–S3.

## 4. Discussion

In this study, we investigated the performance of ADC thresholds using an automated acute stroke lesion delineation algorithm based on assumptions from previous research (8, 15). We tested the algorithm on a large, heterogeneous group of patients incorporating all types and locations of infarcts, representing real-life conditions.

Automatically delineated lesions had significantly lower mean ADC values and volumes than manual delineations. The broader range of mean ADC values in the manual delineations suggests that human raters also delineate tissue with high ADC values, possibly including early vasogenic edema in the delineated areas. The degree to which manually and automatically delineated lesions overlapped varied between patients. In cases where no spatial overlap was observed, the DWI lesions had very low volumes (Figure 5). Factors significantly associated with a higher Dice coefficient and thus better performance of the algorithm were a low DWI lesion ADC value and the lesion size. Our algorithm slightly underperformed commercial automated segmentation algorithms. Previous studies using RAPID^®^ showed a Dice score of 0.52 (*n* = 426) (39) and a mean volumetric difference of −1.95 mL (−12.8–9.0 mL, *n* = 134) when compared to manually delineated DWI lesions (40). Other studies report median Dice scores of 0.39 for optimal, individual DWI thresholds and 0.28 for ADC thresholds when simple thresholding is used (15).

The generalized ADC threshold determined by our ROC analysis is slightly higher than the one used for the algorithm, possibly due to the used ground truth. Individual, non-pooled ROC curves differed significantly between subjects. Reducing the total voxel count for the analysis by narrowing the area of search did not yield better results (Figure 6). A high ADC value in the brain parenchyma and a low ADC value inside the lesion were associated with better diagnostic performance. A higher mean ADC in the surrounding brain parenchyma is likely to enhance contrast and lead to better lesion discrimination when thresholding is used. In our cohort, younger individuals had lower mean ADC values in the DWI lesion and brain parenchyma, the latter being a finding that has been extensively described in the literature (41, 42). This relationship between ADC and age is a possible explanation for the worse performance of individual ADC thresholds in older patients.

As for most radiologic studies evaluating automated image recognition tools, one limitation of our study is the ground truth. As the human rater evaluates the infarcted tissue mainly by assessing the trace images, there are inherent differences in what is picked up by methods using ADC thresholds and what is seen by a human rater. Unlike previous studies, we chose to focus on searching for a threshold to delineate the baseline DWI lesion rather than the “true” ischemic core (8). The reasons for this were twofold - firstly, follow-up imaging required for identifying the true ischemic core was unavailable for many of our patients (30/108). Secondly, the quality of coregistration between follow-up FLAIR images and baseline DWI was, after thorough testing, insufficient for voxelwise comparisons between the two. We nonetheless performed a volumetric comparison between baseline DWI lesions and available follow-up FLAIR lesions (see Supplementary Figure S4). We found that, similar to other studies, both manually and automatically delineated DWI lesions underestimated follow-up FLAIR lesions (43, 44). Image preprocessing is commonly applied as part of MRI segmentation algorithms (45). Precisely how preprocessing is done affects the performance of ADC thresholds. We did not systematically assess the influence of different preprocessing parameters on performance because our goal was not to create a diagnostically superior algorithm but to characterize the diagnostic performance of ADC thresholds in general and investigate the factors that influence it. Though the sample size in our study is considerably larger than in previous studies, it is not large enough for detailed subgroup analysis.

## 5. Conclusion

Our study found that a single, generalized ADC threshold for automated acute infarct delineation does not consistently match an expert’s delineations. Several patient-and lesion-specific factors influence thresholding-based automated segmentation methods and can lead to heterogeneous performance. Our results suggest that research into automated acute infarct delineation should move away from attempting to find a single threshold applicable to all patients. Considering the inherent noisiness of ADC maps, ADC threshold-based automated delineation is particularly unreliable for very small lesions.

## Data availability statement

The datasets presented in this study can be found in the following online repository: https://github.com/goschv/ADC_threshold_study.

## Ethics statement

The studies involving human participants were reviewed and approved by the Institutional Review Board of the Charité Universitätsmedizin, Berlin (EA4/026/08). The study was carried out in accordance with The Code of Ethics of the World Medical Association (Declaration of Helsinki). The patients/participants provided their written informed consent to participate in this study.

## Author contributions

VG: conceptualization, methodology, software, formal analysis, data curation, writing—original draft, writing—review and editing, and visualization. KV, IG, and RG: investigation, and writing—review and editing. SP: formal analysis, writing—review and editing. JF: conceptualization, resources, funding acquisition, and writing—review and editing. AK: conceptualization, methodology, formal analysis, writing—original draft, writing—review and editing, visualization, supervision, and project administration. All authors contributed to the article and approved the submitted version.

## Funding

This project received funding from the Federal Ministry of Education and Research via the grant Center for Stroke Research Berlin (01 EO 0801). We acknowledge financial support from the Open Access Publication Fund of Charité - Universitätsmedizin Berlin and the German Research Foundation (DFG).

## Conflict of interest

JF reports a relationship with Abbvie, AC Immune, Alzheon, Artemida, BioClinica/Clario, Biogen, BMS, Brainomix, Cerevast, Daiichi-Sankyo, EISAI, Eli Lilly, F. Hoffmann-LaRoche AG, Guerbet, Ionis Pharmaceuticals, Janssen, Julius Clinical, jung diagnostics, Merck, Novo Nordisk, Premier Research, Roche, and Tau RxInc that includes: consulting or advisory. AK reports a relationship with Bayer AG that includes: consulting or advisory.

The remaining authors declare that the research was conducted in the absence of any commercial or financial relationships that could be construed as a potential conflict of interest.

## Publisher’s note

All claims expressed in this article are solely those of the authors and do not necessarily represent those of their affiliated organizations, or those of the publisher, the editors and the reviewers. Any product that may be evaluated in this article, or claim that may be made by its manufacturer, is not guaranteed or endorsed by the publisher.
