# Supplementary material for: Automated acute ischemic stroke lesion delineation based on apparent diffusion coefficient thresholds
Source: Front Neurol. 2023 Jul 27;14:1203241. doi: 10.3389/fneur.2023.1203241 (PMC10415099; doi:10.3389/fneur.2023.1203241)
Supplement: Supplementary file 1 [file Data_Sheet_1.docx]

Supplementary Material

Automated acute ischemic stroke lesion delineation based on apparent diffusion coefficient thresholds

**Vitus Gosch^1^, Kersten Villringer, MD^1^, Ivana Galinovic, MD PhD^1^, Ramanan Ganeshan, MD^1^, Sophie K. Piper, PhD^2,3^, Jochen B. Fiebach, MD^1^, Ahmed Khalil, MBBS MSc PGDip MD PhD^1^**

^1^Charité – Universitätsmedizin Berlin, corporate member of Freie Universität Berlin and Humboldt-Universität zu Berlin, Center for Stroke Research Berlin, Berlin, Germany

^2^Charité – Universitätsmedizin Berlin, corporate member of Freie Universität Berlin and Humboldt-Universität zu Berlin, Institute of Medical Informatics, Charitéplatz 1, 10117 Berlin, Germany

^3^Charité – Universitätsmedizin Berlin, corporate member of Freie Universität Berlin and Humboldt-Universität zu Berlin, Institute of Biometry and Clinical Epidemiology, Charitéplatz 1, 10117 Berlin, Germany

*** Correspondence:**Vitus Gosch
vitus.gosch@charite.de

# Supplementary Figures
